# Learning curve analysis of transvaginal pelvic reconstruction surgery with four-wing mesh

**DOI:** 10.3389/fmed.2025.1667340

**Published:** 2026-01-15

**Authors:** Liqiong Huang, Jiajia Zhang, Aijie Xie, Xin Li, Tianjiao Liu, Jinbo Wang, Liwen Song, Aozheng Chen, Jin Qiu

**Affiliations:** 1 Department of Obstetrics and Gynecology, Shanghai Tongren Hospital, Shanghai Jiao Tong University School of Medicine, Shanghai, China; 2 University of Electronic Science and Technology of China, Chengdu Women’s and Children’s Central Hospital, Chengdu, Sichuan, China; 3 West China Second University Hospital, Sichuan University, Chengdu, China

**Keywords:** CUSUM analysis, four-wing mesh, learning curve, surgical proficiency, transvaginal pelvic reconstruction

## Abstract

**Objective:**

Compared to traditional pelvic reconstruction surgery, transvaginal pelvic reconstruction surgery with four-wing mesh (T4) offers significant advantages in reducing postoperative recurrence rates, improving surgical success, and enhancing patients’ quality of life. However, this procedure requires a high level of expertise from the surgeon, and improper use of the mesh may increase the risk of erosion or displacement. The aim of this study was to analyze the learning curve for T4, providing reference for the further promotion and application of this technique.

**Methods:**

A retrospective analysis was conducted on 84 patients with pelvic organ prolapse who underwent the modified T4 procedure between January 2019 and April 2023. Perioperative and follow-up data were collected and compared across different phases of the learning process. The learning curve was plotted using the cumulative sum method, while multiple linear regression analysis was employed to identify factors influencing surgical duration.

**Results:**

All surgeries were successfully completed without the need for conversion to alternative surgical approaches. The overall mean operative time operative time was 47.80 ± 17.18 min. The learning curve revealed three distinct phases: Phase I (exploratory stage, first 20 cases) with a mean operative time of 65.85 ± 18.04 min, Phase II (competence acquisition stage, the next 49 cases) with a mean OT of 44.33 ± 11.28 min, and Phase III (post-learning phase for the final 15 cases, indicating mastery of the T4 procedure) with a mean operative time of 35.07 ± 13.82 min.

**Conclusion:**

The modified T4 procedure demonstrated a three-phase learning curve, with surgical competence appearing to be achieved after approximately 20 cases. Continued experience was associated with improvements in operative efficiency; however, these findings should be interpreted cautiously, as further comparative studies are required to validate the potential clinical advantages of this technique.

## Introduction

Transvaginal pelvic reconstruction surgery has emerged as a highly effective treatment for pelvic organ prolapse (POP), a condition that significantly impacts the quality of life in affected women (1, 2). Among the various surgical techniques, the use of four-wing mesh (T4) has garnered attention due to its potential to improve anatomical outcomes, reduce postoperative recurrence rates, and enhance patient satisfaction (3, 4). The four-wing mesh technique, unlike conventional transvaginal mesh (TVM) systems such as Prolift and Elevate, uses a smaller anatomically adapted mesh with four anchoring arms, thereby reducing dissection, blind punctures, and related complications while enabling more precise fixation.

The T4 technique is applicable not only to anterior vaginal wall prolapse but also to broader indications of pelvic organ prolapse, including rectocele and other multi-compartment defects, thereby serving as a comprehensive approach to pelvic floor reconstruction. This approach, however, presents unique challenges, particularly with regard to surgeon proficiency. As with any technically demanding procedure, the success of transvaginal pelvic reconstruction using T4 is closely tied to the surgeon’s expertise and familiarity with the technique.

Existing research has demonstrated that transvaginal mesh procedures, including those employing the four-wing mesh, are associated with better long-term outcomes compared to traditional methods (5, 6). However, improper mesh placement and handling can result in complications such as mesh erosion, displacement, and infection, leading to suboptimal results and even further surgeries (7–9). These issues underscore the importance of understanding the learning curve associated with this technique to optimize surgical performance and patient safety.

A comprehensive analysis of the learning curve is essential to determine the minimum number of cases required for surgeons to achieve competency in this technique, identify key milestones during the learning process, and establish best practices for training (10). To date, there has been limited research focused specifically on the learning curve of transvaginal pelvic reconstruction with four-wing mesh. Understanding this process is crucial for improving surgical outcomes and reducing the likelihood of complications, particularly in light of the increasing demand for minimally invasive POP surgeries.

This study aims to analyze the learning curve for T4, evaluate the stages of surgeon proficiency, and assess the factors influencing operative success. The findings will provide valuable insights for surgical training programs and the broader clinical application of this technique.

## Materials and methods

### Study design and population

The present study was conducted as part of the Shanghai Longitudinal Cohort Study on pelvic floor reconstruction, an ongoing gynecological cohort study based in Shanghai. This study aims to provide a long-term and detailed follow-up on T4, focusing on identifying the incidence and risk factors for complications such as mesh exposure, infection, and relapse. This sub-cohort analysis was carried out at Shanghai Tongren Hospital, where 84 women undergoing T4 were recruited between January 2019 and April 2023. The pelvic floor reconstruction study, established in April 2022 (ChiCTR2200059282), received approval from the Ethics Committee of Shanghai Tongren Hospital (No. 2021-066-01). And this study was conducted and completed strictly in accordance with the Declaration of Helsinki. Prior to the surgery, all patients were thoroughly informed about the potential risks and benefits, including the risks of intraoperative bleeding, and injuries to the bladder, ureters, and intestines. Written informed consent was obtained from all participants.

Eligible patients included those diagnosed with stage III–IV pelvic organ prolapse involving the anterior vaginal wall, rectocele, or combined defects, for whom the T4 procedure was indicated as a reconstructive treatment. The inclusion criteria for this study were as follows: (i) Patients had stable vital signs and no contraindications for anesthesia or surgery. (ii) Indications for vaginal pelvic reconstruction included stage III or IV pelvic organ prolapse (POP-Q stage), and patients expressed a desire for the procedure. (iii) Based on the preoperative POP-Q score in the D position, a four-wing molding mesh was used if the D point was less than −2. (iv) Patients had no history of abnormal vaginal bleeding or postmenopausal vaginal bleeding, and their Thinprep cytology test (TCT) and human papillomavirus (HPV) tests were normal. (v) For patients with a preserved uterus, there were no malignant lesions detected in the uterus or adnexa. The exclusion criteria were as follows: (i) Patients with systemic diseases that precluded the ability to tolerate surgery. (ii) Patients with malignant tumors. (iii) Virgin patients. (iv) Pregnant patients.

### Standard operating procedures for T4

**FIGURE 1:**
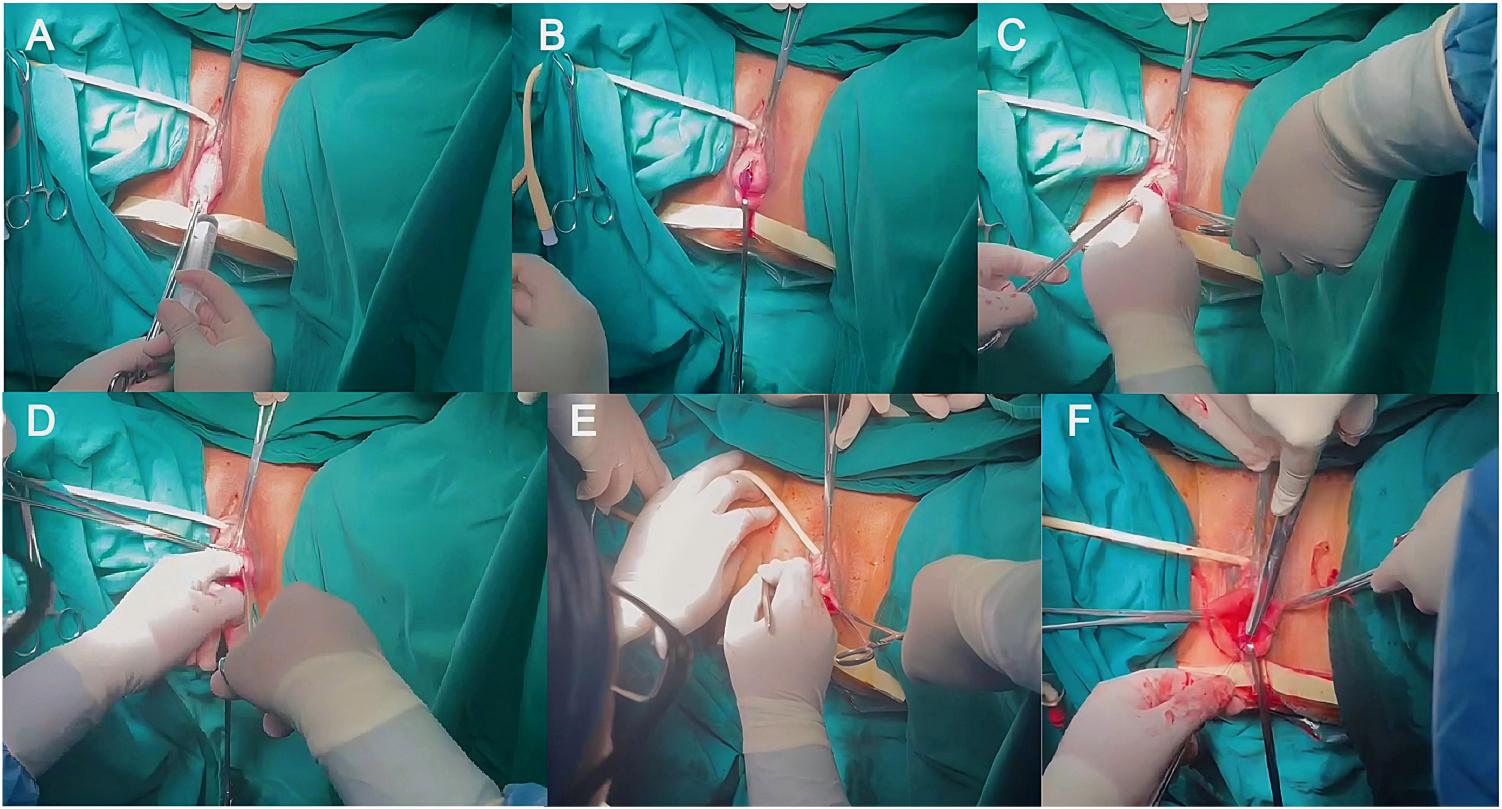
The illustration of the modified six-step procedure for transvaginal pelvic reconstruction with a four-wing mesh. **(A)** Saline is injected into the vesico-vaginal space to create a water pad, which helps to separate the space and prevent bladder injury; **(B)** A scalpel is used to make a full-thickness incision through the vaginal wall until the transparent water pad of the vesico-vaginal space is exposed. **(C–D)** An index finger is employed to bluntly separate the vesico-vaginal space to the level of the bilateral ischiatic spines; **(E)** A 0.3 cm skin incision is made at the level of the bilateral groin-clitoris for the insertion of a shallow mesh band; **(F)** The non-absorbable suture secures the mesh to the top of the anterior vaginal wall and the cervix.

To perform the procedure, use a 20 mL syringe to inject 50 to 60 mL of saline into the vesico-vaginal space, creating a water pad that aids in separating the space and minimizing the risk of bladder injury (Figure 1). Make a 1 cm incision on the anterior wall of the vagina, starting from the distal end of the vesico-cervical attachment and extending to the urethrovesical groove. The procedure was performed using a standardized four-wing mesh (ALPUs Medical Co. Limited, TiLOoPR Total 4 light, 36092).

Using your index finger, bluntly dissect the vesico-vaginal space until reaching the bilateral ischiatic spine level. Create two skin incisions on each side of the perineum: one for the placement of the shallow band and another for the deep band of the mesh.

The first incision, 0.3 cm in length, is made at the bilateral groin-clitoris level for the shallow band. The second incision, also 0.3 cm in length, is positioned 1 cm lateral and 2 cm inferior to the first incision for the deep band. Insert the puncture needle vertically through the first incision from the outside in. Sequentially place the left and right shallow bands through the obturator cavity and the arch tendinous fascia (ATFP) to the anterior vaginal incision. Keep your index finger in the vesico-vaginal space throughout this process as a guide.

Similarly, with the index finger as a guide, insert the deep band of the mesh through the second incision from the outside in, placing the left and right deep bands sequentially through the obturator cavity and ischiatic spine to the anterior vaginal incision. Secure the mesh to the top of the anterior vaginal wall and cervix with non-absorbable sutures, close the incision on the anterior vaginal wall, and adjust the tightness of the bands to ensure proper positioning.

### Data collection

The study collected general characteristics and perioperative data from the patients, including age, height, weight, gravidity, parity, and frequency of vaginal deliveries. Data on the date of surgery, surgical method, operation time, estimated blood loss, and postoperative visual analogue scale (VAS) scores within 24 h were recorded. Additional data included time to flatus post-surgery, postoperative body temperature, preoperative and 48-h postoperative hemoglobin (Hb) levels, whether a urinary catheter was retained, the duration of catheter indwelling, residual urine measurements, length of postoperative stay, and any intraoperative and postoperative complications occurring within one year. The VAS score was utilized to assess postoperative pain, with ratings ranging from 1 to 10 (11). A higher score indicated greater pain severity, with 0 representing no pain and 10 indicating the most severe pain. All procedures were performed by the same surgical team, led by a senior gynecologic surgeon with more than ten years of operative experience in gynecology, including pelvic organ prolapse and vaginal surgery, but with no prior experience in using the four-wing mesh technique before the study period.

### Statistical analysis

Statistical analyses were conducted using SPSS version 25.0 (IBM, Armonk, NY, USA). To compare continuous variables, Kruskal–Wallis tests were employed, while Chi-squared or Fisher’s exact tests were used for categorical data. Multiple linear regression analysis was employed to identify factors influencing surgical duration. All *p*-values were assessed bilaterally, with a significance level set at *p* < 0.05.

In the analysis of the learning curve for T4 surgery, operative time (OT) was utilized as a surrogate marker for surgical competency. The average OT was 47.80 ± 17.18 min. The learning curve was illustrated by plotting the operation number in chronological order on the *x*-axis and the cumulative sum of OT (CUSUM_*OT*_) on the *y*-axis. CUSUM_*OT*_ was calculated as the cumulative sum of the differences between each OT and the average OT (12). Specifically, the CUSUM_*OT*_ for each case was determined by subtracting the average OT from the OT of that case and summing these differences sequentially. For example, the first CUSUM_*OT*_ value was calculated as the OT of the first patient minus the average OT. The second CUSUM_*OT*_ value was the OT of the second patient minus the average OT plus the first CUSUM_*OT*_ value, and so forth. This process continued until the final CUSUM_*OT*_ reached zero. When the CUSUM_*OT*_ demonstrates an upward trend, it signifies that the procedure is in the learning phase. Conversely, a downward trend in CUSUM_*OT*_ indicates that the procedure has reached the proficient stage (13).

## Results

**FIGURE 2:**
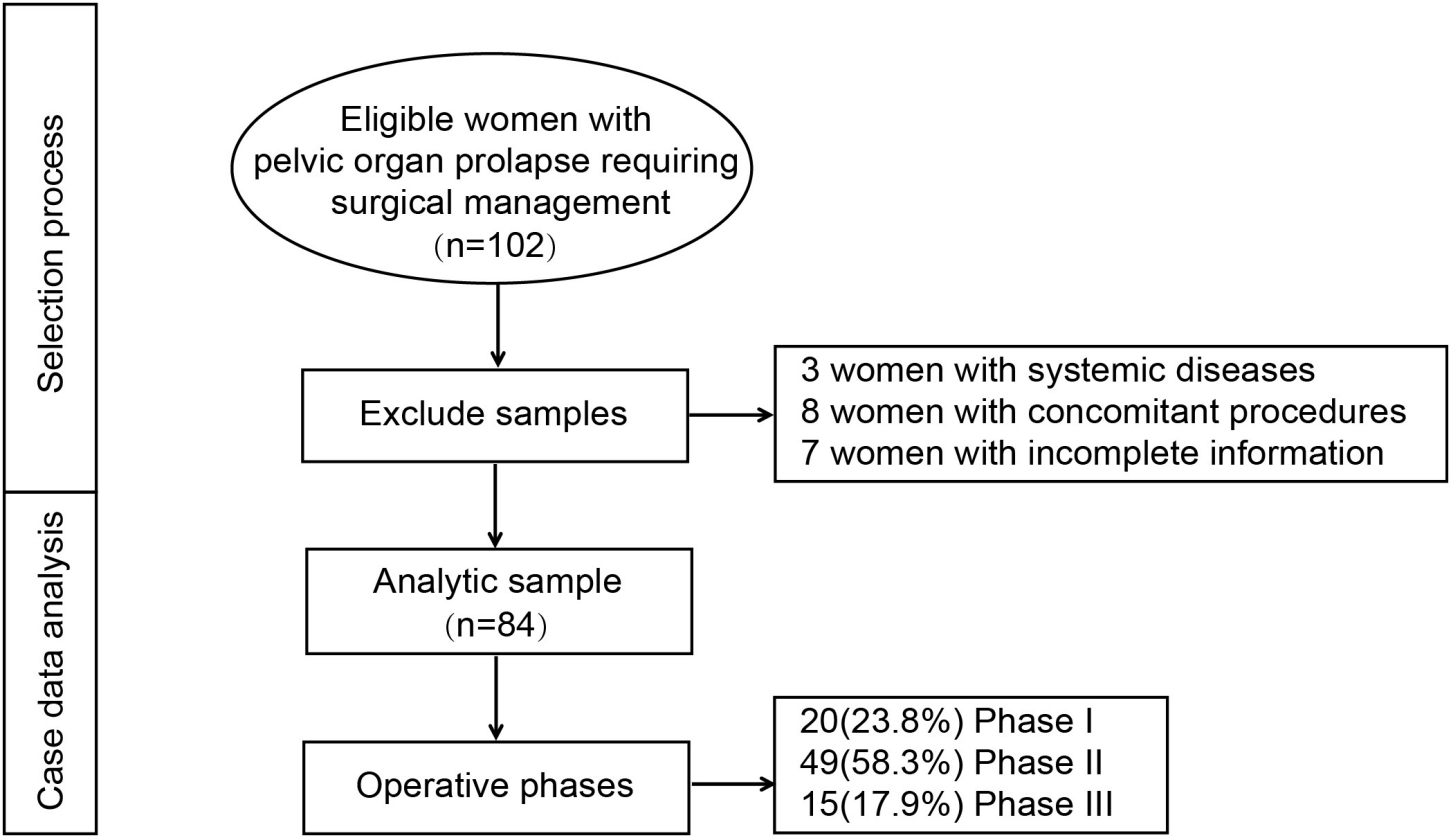
The selection process for this study.

Initially, 102 patients with POP were enrolled in this study (Figure 2). After excluding those with concomitant systemic diseases, concomitant surgeries, or incomplete clinical data, a total of 84 patients were included in the final analysis. All 84 patients were successfully completed without the need for conversion to alternative surgical approaches. Based on the trend of changes in the CUSUM curve, patients were categorized into three stages: the first stage (exploratory stage) included 20 patients, the second stage (competence acquisition stage) included 49 patients, and the third stage (post-learning phase) included 15 patients. Significant differences were observed in surgical time, intraoperative blood loss, and hospitalization duration among these three learning stages. As surgical proficiency improved, there was a noticeable decrease in surgical time, intraoperative blood loss, and length of hospital stay (Table 1).

**TABLE 1:** Characteristics and perioperative data of the patients by phases.

Variables	Phase I *N* = 20	Phase II *N* = 49	Phase III *N* = 15	*p*-value
Age (year)	67.30 ± 9.10	71.71 ± 7.65	63.73 ± 13.92	0.067^c^
BMI (kg/m^2^)	22.71 ± 1.41	23.81 ± 2.67	24.20 ± 3.55	0.186^a^
Parity	1.60 ± 0.88	1.85 ± 7.94	1.86 ± 7.64	0.517^c^
Regular sexual activity	8 (40.0%)	19 (38.8%)	7 (46.7%)	0.861^b^
**Operative information**				
Operative time (min)	65.85 ± 18.04	44.33 ± 11.28	35.07 ± 13.82	< 0.001^a^
Bleeding volume (ml)	71.50 ± 108.20	26.63 ± 13.48	19.67 ± 6.11	0.004^a^
**Post-operative information**				
VAS (day 0)	1.70 ± 0.73	1.91 ± 8.44	2.20 ± 0.41	0.102^c^
VAS (day 1)	1.23 ± 1.39	1.16 ± 1.33	1.93 ± 1.56	0.405^c^
Pain medications	7 (35.0%)	18 (36.7%)	10 (66.7%)	0.095^b^
Catheter placement	20 (100.0%)	49 (100.0%)	12 (80.0%)	0.001^b^
Residual bladder urine (ml)	33.89 ± 28.42	26.03 ± 25.98	19.33 ± 25.32	0.055^c^
Anal exhaust time (hour)	31.40 ± 17.22	29.92 ± 11.42	25.60 ± 6.20	0.370^a^
Hemoglobin decline (g/L)	11.00 ± 8.21	12.12 ± 7.28	9.80 ± 7.72	0.560^c^
Hospital stay (day)	7.90 ± 1.94	6.47 ± 2.19	5.33 ± 1.83	0.002^a^

BMI, body mass index; VAS, visual analog scale.

*^a^*Average and standard deviation. One-way analysis of variance.

*^b^*Number (percentage). Chi-squared test.

*^c^*Average and standard deviation. Kruskal–Wallis test.

There were no complications classified as Grade III to V according to the Clavien–Dindo classification (14). Complications observed included one patient who developed a high fever exceeding 39 °C with cough and expectoration 48 h post-operation. Additionally, three patients experienced urinary tract infections caused by E. coli. One patient had lower extremity venous thrombosis, and another patient suffered a bladder injury, which was possibly related to previous hysterectomy that may have altered pelvic anatomy. Furthermore, one patient developed a deep pelvic hematoma, potentially due to repeated punctures at the same site during the procedure.

**FIGURE 3:**
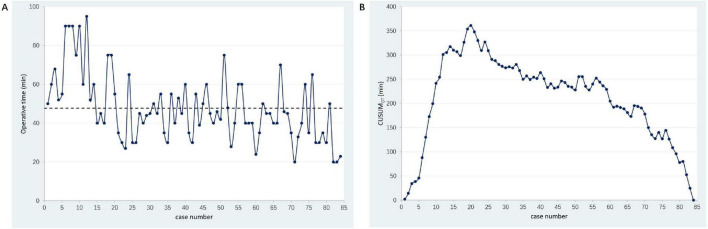
Learning curve analysis of transvaginal pelvic reconstruction surgery with four-wing mesh. **(A)** The relationship between OT and the chronological order of cases; **(B)** The total surgeries into three distinct phases according to the learning curve. The first phase (cases 1–20) is designated as the exploratory stage, with an OT of 65.85 ± 18.04 minutes. The second phase (cases 21–69) represents the acquisition of competence, characterized by a significant reduction in OT to 44.33 ± 11.28 minutes as the surgeon refined the T4 technique. In the third phase (cases 70–84), the surgeon achieved greater proficiency, further reducing the OT to 35.07 ± 13.82 minutes.

The OT serves as an indirect measure of a surgeon’s proficiency with a particular surgical procedure. Figure 3A illustrates the relationship between OT and the chronological order of cases, with an average OT of 47.80 ± 17.18 min. For cases 1 through 20, the OT was above the average. However, from the 21st case onward, improvements in surgical technique led to a rapid decrease in OT. By the 70th case, with increased proficiency in performing T4 surgery, the OT was further reduced. Figure 3B categorizes the total surgeries into three distinct phases according to the learning curve. The first phase (cases 1–20) is designated as the exploratory stage, with an OT of 65.85 ± 18.04 min. The second phase (cases 21–69) represents the acquisition of competence, characterized by a significant reduction in OT to 44.33 ± 11.28 min as the surgeon refined the T4 technique. In the third phase (cases 70–84), the surgeon achieved greater proficiency, further reducing the OT to 35.07 ± 13.82 min.

Figure 1 illustrates the modified six-step procedure for T4. Saline is injected into the vesico-vaginal space to create a water pad, which helps to separate the space and prevent bladder injury; A scalpel is used to make a full-thickness incision through the vaginal wall until the transparent water pad of the vesico-vaginal space is exposed. An index finger is employed to bluntly separate the vesico-vaginal space to the level of the bilateral ischiatic spines. A 0.3 cm skin incision is made at the level of the bilateral groin-clitoris for the insertion of a shallow mesh band. The non-absorbable suture secures the mesh to the top of the anterior vaginal wall and the cervix.

Multivariate linear regression analysis was conducted to further investigate the factors influencing operation time (OT). After adjusting for age and parity, the analysis revealed significant associations between OT and both BMI and learning phases. Specifically, each 1 kg/m^2^ increase in BMI was associated with an approximate increase of 2.9 min in OT (95% CI: 1.51, 4.31; *p* = 0.033). Conversely, advancing one level in the learning phase was associated with a reduction in OT of about 12.4 min (95% CI: −18.52, −6.22; *p* = 0.008) (Table 2).

**TABLE 2:** Association between operative time and perioperative characteristics.

Variables	Beta	95% CI	*P*-value	VIF
***R*^2^ = 0.240**				
Age (year)	2.35	(−1.52, 6.22)	0.410	1.273
BMI (kg/m^2^)	2.91	(1.51, 4.31)	0.033	1.154
Parity	−1.79	(−4.92, 1.34)	0.229	1.095
Phase (I/II/III)	−12.37	(−18.52, −6.22)	0.008	1.219

BMI, body mass index.

## Discussion

The learning curve associated with T4 is critical for understanding how surgical proficiency evolves over time and its impact on operative outcomes. This study’s analysis provides valuable insights into the stages of learning, the factors influencing surgical performance, and the implications for clinical practice.

Our study identified three distinct phases in the learning curve for T4 surgery. Initially, during the exploratory phase (cases 1–20), the average OT was significantly higher (65.85 ± 18.04 min) compared to subsequent phases. This extended OT reflects the learning process, where surgeons are adapting to new techniques and gaining familiarity with the procedure. The significant reduction in OT to 44.33 ± 11.28 min in the competence acquisition phase (cases 21–69) indicates that surgeons rapidly acquire proficiency as they gain experience. Finally, in the post-learning phase (cases 70–84), further improvement in surgical efficiency is evident, with the OT decreasing to 35.07 ± 13.82 min. This phase highlights the stabilization of skills and the attainment of a higher level of expertise, leading to more efficient and effective surgery. Shorter operative time not only reflects improved surgical proficiency but also reduces intraoperative blood loss, lowers the risk of anesthesia-related complications, and contributes to faster postoperative recovery and shorter hospital stay.

The prolonged hospitalization in the initial phase was primarily related to conservative postoperative management, as surgeons required a five-day observation with delayed catheter removal to ensure safe recovery. With increased experience, the postoperative protocol was optimized to a 3-day observation, resulting in shorter hospital stays without compromising patient safety.

Multivariate linear regression analysis identified BMI and learning phases as significant factors influencing OT. Specifically, a 1 kg/m^2^ increase in BMI was associated with a 2.9-min increase in OT. This finding suggests that higher BMI may complicate the surgical procedure, possibly due to increased tissue thickness or the challenge of maneuvering in a confined surgical space (15, 16). Addressing these challenges through tailored surgical techniques or preoperative optimization might help mitigate the impact of BMI on OT.

Moreover, in light of the observed complications, several intraoperative precautions warrant emphasis. To minimize the risk of bladder injury, careful blunt dissection of the vesico-vaginal space and the use of finger guidance during mesh arm placement are critical. Strict aseptic technique and timely removal of the urinary catheter can help reduce urinary tract infections, while avoiding repeated punctures at the same site may decrease the likelihood of pelvic hematoma. In addition, early mobilization and appropriate perioperative thromboprophylaxis are essential to lower the risk of venous thromboembolism.

Conversely, advancing through the learning phases was associated with a significant reduction in OT. Each progression in the learning phase corresponded to a 12.4-min decrease in OT, underscoring the importance of experience in refining surgical skills. This reduction is likely due to increased familiarity with the surgical anatomy, improved technique, and enhanced efficiency in performing the procedure. These results emphasize the need for structured training programs and hands-on practice to accelerate the learning process and achieve optimal surgical outcomes (17).

The identification of distinct learning phases and the impact of factors on OT have several implications for clinical practice. Firstly, understanding the learning curve can help in setting realistic expectations for surgical outcomes and planning appropriate training and support for novice surgeons. It is crucial for institutions to recognize the need for a defined training period where less experienced surgeons may require additional supervision and guidance. Secondly, the data from this study underscores the importance of continuous learning and adaptation in surgical practice. Surgeons should be encouraged to engage in ongoing education and skill development to maintain and improve their proficiency.

T4 surgery is a complex gynecological procedure that presents several challenges for pelvic floor specialists (18–20), including: (1) difficulties and risks associated with separating the bladder and cervical spaces, which can lead to bleeding; (2) the potential for inaccurate positioning and safety concerns due to blind punctures; (3) issues related to mesh exposure and shrinkage; (4) the risk of bladder or rectal injury; and (5) the problem of recurrence. In this study, the modified T4 technique was developed to address these issues. This modification involved detailed adjustments to traditional practices, aiming to improve safety and effectiveness in performing the procedure.

First, the “water pad” technique was introduced to prevent bladder injury: Traditional surgeries often involve making a reverse “T” incision in the vaginal wall. In contrast, the improved technique involves injecting 50–60 mL of normal saline into the vesico-vaginal space using a 20 mL syringe before making any incisions. This technique creates a water pad that helps to separate the vaginal wall from the bladder, reducing the risk of bladder injury. To avoid accidental bladder puncture during injection, the depth of injection is adjusted by palpating the bladder’s position.

Second, advancements were made in the method of vaginal wall incision: Traditionally, the vaginal wall is cut layer by layer, with bleeding controlled by electrocoagulation using an electric knife. The modified procedure involves making a direct incision through the entire vaginal wall with a scalpel until the transparent vesico-vaginal space is exposed. This approach avoids the vascular network in the vaginal wall, which reduces bleeding and eliminates the need for electrocoagulation. By preserving the vaginal wall’s blood supply, this technique promotes better healing and reduces the likelihood of mesh exposure.

Third, the fixation technique for the “shallow band” of the mesh was refined: In traditional procedures, the shallow band of the mesh is often fixed near the urethral opening to address anterior wall prolapse. In the improved technique, the shallow band is fixed below the cervix vesicae. It is crucial that the shallow band is positioned at least 3 cm below the urethral opening. The position of the cervix vesicae is verified by palpating the balloon with the catheter, which helps to avoid postoperative urinary retention.

Fourth, advancements in the separation techniques for the vesico-vaginal space and improvements in the puncture techniques for the “deep branch”: One of the primary challenges in transvaginal mesh surgery is the “blind” nature of the procedure under non-visual conditions. Traditionally, scissors are used to separate the space between the bladder and vagina to reach the area around the ischial spine, with a drag hook employed to protect the bladder. In the modified technique, the index finger is utilized to separate the space and palpate the ischial spine, guiding the puncture device. The finger technique involves “entering the pelvis laterally from the ischial spine and moving upward,” which helps protect the bladder during puncture.

Fifth, refinement in mesh fixation methods: The traditional approach uses a 6-point suture method to secure the mesh to the cervix and anterior vaginal wall. The improved method involves a 2-point fixation technique: the upper edge of the mesh is fixed to the lower urethrovaginal transverse groove, and the lower edge is fixed to the distal cervical end. This 2-point fixation facilitates a smoother and more complete unfolding of the mesh within the bladder and cervix space, reducing the risk of wrinkling, mesh exposure, and prolapse recurrence.

Sixth, enhancements in closing techniques for the vaginal walls: Traditionally, a significant portion of the anterior vaginal wall is removed and then sutured. In the improved technique, no excess vaginal wall tissue is removed, regardless of its size, to ensure that the mesh has adequate space to expand and to avoid vaginal contracture pain. These modifications have collectively reduced the complexity of TVM surgery, significantly shortened the operation time, and decreased intraoperative blood loss.

The modified T4 technique should be regarded as a refinement of Prolift-based methods, with innovative features including shallow band repositioning, finger-guided puncture, and preservation of vaginal wall tissue. These changes, though incremental, provide meaningful improvements in operative safety, efficiency, and postoperative outcomes, thereby offering clinical advantages beyond the conventional Prolift procedure.

While this study provides valuable insights, it has limitations. The sample size and the single-center design may limit the generalizability of the findings. Future research should include larger, multi-center cohorts to validate these results and explore additional factors influencing the learning curve. Moreover, assessing long-term outcomes and patient satisfaction associated with different stages of the learning curve could further enrich our understanding of the impact of surgeon experience on clinical outcomes.

## Conclusion

Our data identified three distinct phases in the T4 learning curve, suggesting that an experienced surgeon may achieve basic surgical competence after approximately 20 cases. As experience with the procedure increases, operative time and perioperative parameters appear to improve; however, these findings should be interpreted with caution in the absence of direct comparative data, and further controlled studies are needed to confirm any potential advantages of the modified T4 technique.

## Data Availability

The original contributions presented in this study are included in this article/supplementary material, further inquiries can be directed to the corresponding authors.
